# A Large French Case-Control Study Emphasizes the Role of Rare *Mc1R* Variants in Melanoma Risk

**DOI:** 10.1155/2014/925716

**Published:** 2014-04-10

**Authors:** Hui-Han Hu, Mériem Benfodda, Nicolas Dumaz, Steven Gazal, Vincent Descamps, Agnès Bourillon, Nicole Basset-Seguin, Angélique Riffault, Khaled Ezzedine, Martine Bagot, Armand Bensussan, Philippe Saiag, Bernard Grandchamp, Nadem Soufir

**Affiliations:** ^1^INSERM U976, Centre de Recherche sur la Peau, Hôpital Saint Louis, 1 Avenue Claude Vellefaux, 75010 Paris, France; ^2^Laboratoire de Génétique, Hôpital Bichat Claude Bernard, APHP, IFR02, Université Paris 7, 46 rue Henri Hucahrd, 75018 Paris, France; ^3^UMR S738, Faculté de Médecine Xavier Bichat, 16 rue Henri Huchard, 75018 Paris, France; ^4^Service de Dermatologie, Hôpital Bichat Claude Bernard, APHP, Université Paris 7, 46 rue Henri Hucahrd, 75018 Paris, France; ^5^Service de Dermatologie, Hôpital Saint Louis, APHP, Université Paris 7, 1 Avenue Claude Vellefaux, 75010 Paris, France; ^6^Service de Dermatologie, CHU Bordeaux, Université V Segalen Bordeaux 2, 12 rue Dubernat 33404 Bordeaux, France; ^7^INSERM U876, 146 rue Leo Saignat, 33076 Bordeaux, France; ^8^Service de Dermatologie, Hôpital Ambroise Paré, APHP, 9 Avenue Charles-de-Gaulle, 92100 Boulogne Billancourt, France; ^9^Université Saint Quentin en Yvelines, 55 Avenue de Paris, 78000 Versailles, France

## Abstract

*Background*. The *MC1R* gene implicated in melanogenesis and skin pigmentation is highly polymorphic. Several alleles are associated with red hair and fair skin phenotypes and contribute to melanoma risk. *Objective*. This work aims to assess the effect of different classes of *MC1R* variants, notably rare variants, on melanoma risk. *Methods*. *MC1R* coding region was sequenced in 1131 melanoma patients and 869 healthy controls. *MC1R* variants were classified as RHC (*R*) and non-RHC (*r*). Rare variants (frequency < 1%) were subdivided into two subgroups, predicted to be damaging (*D*) or not (*nD*). *Results*. Both *R* and *r* alleles were associated with melanoma (OR = 2.66 [2.20–3.23] and 1.51 [1.32–1.73]) and had similar population attributable risks (15.8% and 16.6%). We also identified 69 rare variants, of which 25 were novel. *D* variants were strongly associated with melanoma (OR = 2.38 [1.38–4.15]) and clustered in the same *MC1R* domains as *R* alleles (intracellular 2, transmembrane 2 and 7). *Conclusion*. This work confirms the role of *R* and *r* alleles in melanoma risk in the French population and proposes a novel class of rare *D* variants as important melanoma risk factors. These findings may improve the definition of high-risk subjects that could be targeted for melanoma prevention and screening.

## 1. Introduction 

The incidence of cutaneous melanoma, the most lethal type of skin cancer, is increasing in western countries, doubling every ten years [1]. Melanoma is a complex disease that arises through multiple etiological pathways [2]. Ultraviolet radiation exposure is the main environmental cause, and pigmentation characteristics such as light skin, hair and eye colour, and high number of nevi have also been identified as melanoma risk factors [3].

Germline mutations in high-penetrance melanoma predisposing genes* CDKN2A* and* CDK4* have been found in 20% of familial melanoma cases [4]. Recently, a third major gene,* BAP1,* that predisposes to melanoma (mainly ocular), mesothelioma, and possibly additional cancers has been identified [5–7]. In addition, numerous low penetrant susceptibility variants, which modulate melanoma risk, have also been described. These genes are mainly involved in melanogenesis (*MC1R, ASIP, TYR, TYRP1*, and* SLC45A2*) and melanocyte differentiation (*MITF, KIT*, and* EDNRB*) [8–12].Recently, variants in other pathways, such as DNA repair, genome maintenance integrity, and immunological pathways (*TERT-CLPTM1*,* CASP8*,* ATM,* and* MX2*), have also been linked to melanoma predisposition [13–17].

Among pigmentation genes,* MC1R*, which is the most studied, is associated with human skin pigmentation and melanoma susceptibility. MC1R, the receptor for *α*-melanocyte stimulating hormone (*α*-MSH), is a G protein coupled receptor with seven transmembrane domains that regulates the relative concentration of brown-black eumelanin and red-yellow pheomelanin [18]. Eumelanin has been shown to reduce the accumulation of DNA photodamage and to protect melanocytes from UV-induced apoptosis. Pheomelanin is, on the contrary phototoxic, generating oxidative stress by the production of reactive oxygen species [19–21].

*MC1R* is highly polymorphic within Caucasian populations [21]. A recent review has documented 57 nonsynonymous and 25 synonymous polymorphisms in different populations [22]. RHC (red hair color) variants (also called “*R*” alleles) lead to nonfunctional or diminished functional receptors [23], preferentially induce pheomelanin production, and are therefore associated with red hair, light skin, poor tanning ability, and heavy freckling [24–27]. Other* MC1R* variants that impact less strongly on MC1R function are called non-RHC variants and are labelled “*r*” alleles.

Numerous association studies have demonstrated the important role of* MC1R R* variants in melanoma predisposition [8, 11, 28, 29]. The influence of* MC1R r* variants on melanoma risk has also been reported but there are several discrepancies in the different published works [4, 29–31]. In addition, to date there is no conclusion on the role of rare* MC1R* variants in melanoma. In this large French case-control study, we investigated the role of different classes of* MC1R* variants in melanoma risk, focussing particularly on the role of rare* MC1R *variants.

## 2. Patients and Methods

### 2.1. Studied Populations

A cohort of 1131 Caucasian melanoma patients was recruited between 2002 and 2008 from the dermatology departments of all university-affiliated hospitals in Paris (the MelanCohort). The main characteristics of the patients have been previously described [32]. Melanoma was sporadic in 784 patients (69%), including 81 patients (7%) who had multiple melanomas and 229 patients (20%) who had familial melanoma (at least 2 cases in first- or second-degree relatives, including the proband). Among the familial and multiple sporadic cases, 8.5% of patients carried mutations in the* CDKN2A* or* CDK4* gene.

The control group comprised 869 ethnically matched skin cancer-free blood donors recruited from the EFS (Etablissement Français du Sang) in Bichat and Saint-Louis hospitals over the same period. These subjects have previously been described and used as controls in case-control studies [32–34].

Data from patients and controls regarding sex, age, ethnic origin, date of birth, anatomoclinical data, pigmentation characteristics (hair, eye, and skin colours), skin type (Fitzpatrick classification I to IV), nevus count (<10, 10–50, 51–100, and >100), and heavy freckles (yes/no) were collected in a standard document. Ancestry was investigated through birth location of parents and grandparents, and only those with a Caucasian ancestry were retained for the study. The pigmentation characteristics of patients and controls are summarized in Supplementary Table 1 (see Supplementary Materials available online at http://dx.doi.org/10.1155/2014/925716). All subjects signed an informed consent form and provided a blood sample.

### 2.2. Sequencing and Mutational Analysis

The coding sequence of* MC1R* deposited in GenBank (NM_002386.3) was amplified with 2 couples of primer selected by UCSC Genome Browser Gateway (data available upon request) in 1131 melanoma patients and 869 controls. The PCR mix contained 20 ng of genomic DNA, 2.5 mM MgCl_2_, 50 *μ*M of each dNTP, 400 nM of each primer, and 0.5 U Ampli TaqGold polymerase (Applied Biosystems, Courtaboeuf, France). A 62°C annealing temperature was used for PCR amplification. PCR products were verified on a 2% agarose gel and purified by EXOSAP-IT (USB Corporation, OH, USA). Sequencing reaction was performed on 8900 Fast Thermal Cycler (Applied Biosystems), using 10 ng of purified PCR products and the Big-Dye Terminator Cycle Kit (Applied Biosystems). Sequence analysis was performed with an ABI-Prism 3130 automated sequencer (Applied Biosystems) and read with SeqScape software v2.5 (Applied Biosystems).

### 2.3. Classification of* MC1R* Variants

The functional impact of numerous* MC1R* variants has been assessed in previous studies [47] (Supplementary Table 2). Some variants lead to poor MC1R expression, due to endoplasmic reticulum (ER) retention or aberrant trafficking from ER to Golgi [50]. Other variants result in a diminished functional receptor due to lower affinity for *α*-MSH, reduced coupling with cAMP, or decreased ability to stimulate cAMP production [47, 50–53].

In order to predict the impact on protein function of* MC1R* variants, we used* in silico* prediction tools, SIFT (http://sift.jcvi.org/), SNPs3D (http://www.snps3d.org/), and PolyPhen (http://genetics.bwh.harvard.edu/pph/). Rare variants, which were defined by an allele frequency <1% and predicted to be damaging by at least 1 of 3 prediction tools, were predicted to be damaging (*D*) variants, while variants without any damaging effect were regarded as nondamaging (*nD*) variants. Variants that had a clear functional impact (i.e., nonsense or frameshift mutations) were classified as damaging (*D*) variants.

### 2.4. Statistical Analysis

Statistical analysis was performed by using PASW software version 18. The level of significance for all tests corresponded to an alpha error rate of 5%. All odds ratios (OR) were calculated with 95% confidence intervals.

To assess the association of *R* and *r* variants with melanoma, we used Fisher's exact test, the number of haplotypes without any variants alleles (wild-type and synonymous variants) being considered as reference. Rare *r* variants were thereafter classified into two subgroups according to functional prediction (predicted to be damaging (*D*) or nondamaging (*nD*)), and their effects on melanoma risk were also tested.

A multivariate analysis adjusted for hair and eye colours, skin type, and nevus count was carried out to investigate the independent effect of *R* and *r* alleles on melanoma risk. *P* values and their corresponding OR were calculated with logistic regression. Due to the interdependency of all our tests and the magnitude of our results, no correction for multiple testing was performed.

In order to quantify the impact of* MC1R R*, *r*, and *D* variants on melanoma risk, their population attributable fractions (PAF) were calculated as follows: PAF = (*p* × (OR − 1)/(*p* × (OR − 1) + 1), where *p* is the proportion of controls carrying the risk alleles [34].

Finally, in order to investigate the respective role of MC1R protein domains in melanoma, the number of *D* and *nD* variants in different domains was compared between patients and controls. Each protein domain was determined by ExPASy Bioinformatics Resource Portal (http://expasy.org/) using UniProtKB Q01726.2 as query.

## 3. Results

### 3.1. Characterization of* MC1R* Variants

**Figure 1:**
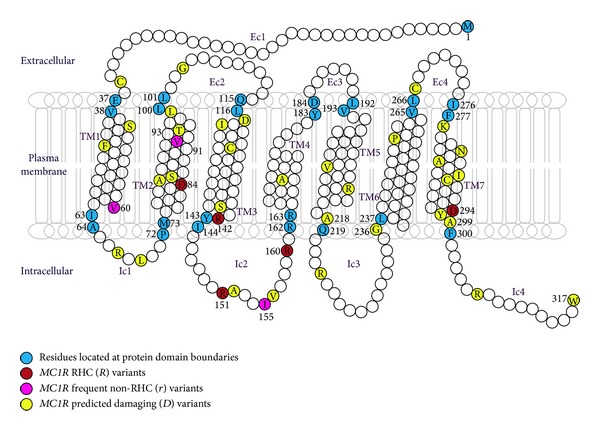
Structure of human melanocortin-1 receptor. Putative structure is drawn according to the two-dimensional model of Ringholm et al., 2004 [54]. The amino acid sequence corresponds to the wildtype consensus (UniProtKB Q01726.2). Each protein domain is framed by residues marked in blue. Residues with *R*, frequent *r*, and predicted-to-be-damaging (*D*) alleles are shown in red, pink, and yellow, respectively. Ec: extracellular domain; Ic: intracellular domain; TM: transmembrane domain; *R*: RHC alleles; *r*: frequent non-RHC alleles; *D*: predicted damaging variants.

By sequencing the entire* MC1R* coding sequence, we found 79* MC1R *variants: 2 nonsense, 3 frameshift, 53 missense, and 21 silent variants (Table 1), 9% of which were localized in the extracellular portion of the receptor, 65% in the transmembrane domains, and 25% in the cytoplasmic domains (Figure 1).

Five *R* alleles (D84E, R142H, R151C, R160W, and D294H) and 5 frequent *r* alleles (V60L, V92M, I155T, R163Q, and T314T) were found in our cohorts. In addition, we found 69 rare *r* alleles, consisting of 44 missense, 2 nonsense, 3 frameshift, and 20 silent variants. Interestingly, 25 were novel* MC1R* variants, 15 of which (52%) were predicted to have a functional impact (*D* variants). Amongst the 69 rare *r* alleles, 40 (58%) were predicted to be *D* variants, including 35 missense, 2 nonsense, and 3 frameshift variants (Table 1).

### 3.2. Association of Different* MC1R* Variant Subgroups with Melanoma Risk

The association of different subgroups of* MC1R* variants with melanoma risk was assessed as described in Section 2. Collectively, *R* alleles were strongly associated with melanoma (OR = 2.66 [2.20–3.23]; *P* = 2.69*E* − 25) (Table 2). In addition, association of individual *R* alleles with melanoma was significant (ORs = from 2.33 to 2.9) for all *R* variants except for D84E (*P* = 0.092). We calculated an estimated PAF due to *R* variants of 15.8%. By decreasing order, the PAFs were, respectively, 7.4 (R151C), 5.7 (R160W), 2.9 (D294H), 1 (R142H), and 0.8 (D84E) (Table 2).

Interestingly, *r* alleles were also associated, although less strongly, with melanoma (OR = 1.51 [1.32–1.73]; *P* = 1.40*E* − 09) (Table 2). In addition, three frequent *r* alleles (V60L, V92M, and I155T) were associated individually with melanoma, but the association for the R163Q variant did not reach significance (*P* = 0.087). Of note, the I155T variant associated almost as strongly as *R* alleles with melanoma (OR = 2.61 [1.35–5.12]) and the other frequent *r* alleles conferred risks equal to roughly half the risk of *R* alleles (ORs = from 1.47 to 1.73). The PAF of *r* variants was very similar to that of *R* variants (16.6%). By decreasing order, the PAFs were, respectively, 6.5 (V60L), 4.5 (V92M), 1.3 (I155T), and 1.2 (R163Q) (Table 2).

Furthermore, rare *r* variants were also strongly associated with melanoma (OR = 1.92 [1.20–3.07]; *P* = 0.004) (Table 2). We divided rare *r* alleles into *D* and *nD* subgroups according to* in silico* functional predictions. Interestingly, *D* variants were associated with melanoma susceptibility as strongly as *R* alleles (OR = 2.38 [1.38–4.15]; *P* = 0.001). Of note, the estimated PAF of *D* variants was 1.6% and contributed almost completely to the PAF of rare *r* variants (Table 2). On the contrary, *nD* variants had no impact on melanoma susceptibility (*P* = 1). Moreover, the average age at melanoma diagnosis of patients carrying *D* variants was younger than that of patients without *D* variants (45 and 54, resp.; *P* = 0.04). Finally, *D* variants, like *R* variants, were also associated with familial melanoma (OR = 4.78).

To investigate whether the effect of the two main* MC1R* variant categories on melanoma susceptibility was independent of pigmentation traits, we conducted a multivariate analysis including the main clinical melanoma risk factors using logistic regression (Tables 3(a) and 3(b)). This showed a persistent role of *R* and *r* alleles on melanoma risk (respective ORs = 2.22 [1.66–2.97] and 1.26 [1.04–1.52]).

### 3.3. Impact of the Different MC1R Protein Domains

In order to study the impact of MC1R protein domains on melanoma predisposition, the different classes of* MC1R* variants (*R*, *r*, *D*, and *nD*) were positioned on the different protein domains (Figure 1).

Most* MC1R *variants were localized in six receptor domains: transmembrane 1, 2, 3, and 7, intracellular 2, and C terminal domains. Very few variants were located in the extracellular portion. Among these domains, variants located in the intracellular domain 2 and in the transmembrane domain 7 had the highest impact on melanoma risk (OR = 2.75 [2.22–3.40] and OR = 2.48 [1.67–3.69]).

The repartition of *R*, *r*, *D*, and *nD* variants in each protein domain indicated in Table 4 showed that 63% of *D* variants were located in four domains (intracellular 2 and transmembrane 2, 5, and 7) whereas only 18% of *nD* variants were located in these domains (*P* < 0.0001). Importantly, three of these four domains also contain at least one *R* variant, suggesting an important role of these domains in MC1R function and pathogenicity.

## 4. Discussion

*MC1R* variants are usually classified into two main categories, RHC (*R*) and non-RHC (*r*), according to their association or not with the red hair colour phenotype [21, 24, 27, 55]. For the past ten years several association studies have demonstrated the importance of *R* alleles on melanoma predisposition [28, 29, 42, 56, 57]. However, the influence of rare* MC1R *variants on melanoma predisposition has been poorly investigated which prompted us to study in detail the role of these variants in the French population.

In this study, we confirmed the association of most *R* alleles and melanoma risk with strengths that were close to those observed in previous studies [28, 29, 42, 56, 57].

We also showed a clear association of frequent* MC1R r* alleles, especially V60L, V92M, and I155T, with melanoma. In an early meta-analysis neither of them was found to be associated with melanoma [55], whereas, in a more recent and larger meta-analysis, both were associated with melanoma [57]. V60L is a loss of function variant with reduced coupling to the cAMP signalling [50, 53] and V92M has a lower affinity for *α*-MSH than wild-type MC1R and a decreased ability to stimulate the production of cAMP [23]. These functional data argue for an association of theses variants with melanoma risk.

Even though the OR of *R* alleles was much higher than that of *r* alleles, their PAFs were very close (15.8% for *R* and 16.6% for *r*) suggesting that the impact of *r* alleles on melanoma seems to be important in the French population. Notably, in our work, the individual PAFs of V60L and V92M (6.5% and 4.5%) were very close to that of *R* alleles R151C and R160W (7.4% and 5.7%). Our results are different from that published by Williams et al., in which the PAF of *r* variants is only 7.4. This may be due at least, in part, to the high allelic frequency of *r* variants observed in French melanoma patients (0.43) (versus 0.33 in Williams meta-analysis) [57]. This further emphasises the role *r* alleles in melanoma risk according to the ethnical background of the population studied. In addition, both *R* and *r* alleles remained associated with melanoma risk in multivariate analyses, suggesting that they exert a role in melanoma risk independently of their effect on pigmentation, as previously suggested.

In this work we identified 25 variants that have not been reported before [22, 23, 31, 47], further underscoring the highly polymorphic character of* MC1R*. According to Gerstenblith's work, the proportion of rare* MC1R* variants varies across populations and within Caucasian populations [23]. In our study, the proportion of rare variants (87%) was close to that described in Scherer's work (74% in Germany and 78% in Spain) [31]. Interestingly, rare variants predicted to be damaging (*D* variants) were associated with melanoma as strongly as *R* alleles (OR = 2.38 [1.38–4.15]) whereas variants not predicted to be damaging (*nD*) had no effect on melanoma. In addition, PAF of *D* variants (1.6%) was higher than two *R* variants (R142H and D84E), which seems to indicate that the contribution of this subgroup in melanoma predisposition should be taken into consideration, at least in the French population.

Yet, there is a limitation in our work: the absence of functional studies concerning the potential effects of these novel* MC1R* variants, notably on *α*-MSH binding, receptor cellular localisation, and cAMP signalling.

Finally, the majority of *D* variants were located in the same domains as the *R* alleles (intracellular 2 and transmembrane 2, 5, and 7, Table 4 and Figure 1). It had been shown before that there was a similar localization of *D* variants in the German, Spanish, and Italian populations [29, 31] suggesting an important role of these domains in the receptor's function.

## 5. Conclusion

In this large study we confirmed the role of* MC1R R* alleles in melanoma susceptibility and clearly showed that* MC1R r* alleles also significantly increase the risk of melanoma. In addition, we defined the role of rare* MC1R* variants and proposed a novel class of *D* variants that are strong melanoma risk factors. These findings might help in the definition of high-risk melanoma subgroups that could be targeted for melanoma prevention.

## Supplementary Material

Supplementary Table 1: Pigmentation characteristics, which include hair, eye, and skin color, skin type, nevus count, and heavy freckles, were collected from patients and controls studied in this work.
Supplementary table 2: Functional impact of all *MC1R* variants, which have been investigated to date, was collected from many different studies and summarized in this table.

## Figures and Tables

**Table 1:** All variants found in the study.

Nucleotide change	Amino acid change	Number of chromosome		Predicted to be damaging (*D*) variant^a^	Rare variant^b^	Novel variant^c^	Reference
		Patients (chr = 2262)	Controls (chr = 1738)				
Nonsynonymous							
c.100C > T	p.R34W		1	No	Yes	**Yes**	
c.104G > A	p.C35Y	1		Yes	Yes	No	[35]
c.112G > A	p.V38M	2	3	No	Yes	No	[35]
c.122C > G	p.S41C		2	Yes	Yes	**Yes**	
c.133T > C (rs61996344)	p.F45L	2		Yes	Yes	No	[36]
**c.178G > T (rs1805005)**	**p.V60L**	359	254	Yes	No	No	[25]
c.199C > T	p.R67W		2	Yes	Yes	No	[37]
c.200G > A (rs34090186)	p.R67Q	1	1	No	Yes	No	[38]
c.205C > G	p.L69V		1	Yes	Yes	**Yes**	
c.241G > C	p.A81P	1		Yes	Yes	No	[24]
c.247T > C (rs34474212)	p.S83P	5	1	Yes	Yes	No	[39]
c.252C > A (rs1805006)	p.D84E	23	13	RHC	No	No	[27]
**c.274G > A **	**p.V92M**	188	113	Yes	No	No	[27]
c.284C > T (rs34158934)	p.T95M	6	2	Yes	Yes	No	[27]
c.296T > C	p.L99P	1		Yes	Yes	**Yes**	
c.310G > A (rs2229617)	p.G104S		1	Yes	Yes	No	[24]
c.350A > T	p.D117V	1		Yes	Yes	**Yes**	
c.359T > C (rs33932559)	p.I120T	1		Yes	Yes	No	[35]
c.364G > A	p.V122M	1	4	No	Yes	No	[40]
c.373T > C	p.C125R	1		Yes	Yes	No	[41]
c.389C > T	p.S130F	1		Yes	Yes	**Yes**	
c.415G > A	p.A139T	1		Yes	Yes	No	[31]
c.417G > A	p.V140M	1		No	Yes	**Yes**	
c.419T > G	p.V140G		1	Yes	Yes	**Yes**	
c.424C > T	p.R142C	1		Yes	Yes	No	[42]
c.425G > A (rs11547464)	p.R142H	29	13	RHC	No	No	[25]
c.451C > T (rs1805007)	p.R151C	211	76	RHC	No	No	[25]
c.456C > A	p.Y152X	1		Yes	Yes	No	[43]
**c.464T > C (rs1110400)**	**p.I155T**	35	14	Yes	No	No	[25]
c.467T > C	p.V156A		1	Yes	Yes	No	[31]
c.478C > T (rs1805008)	p.R160W	152	59	RHC	No	No	[25]
c.479G > A	p.R160Q	2		Yes	Yes	No	[36]
**c.488G > A (rs885479)**	**p.R163Q**	75	57	Yes	No	No	[25]
c.512C > A	p.A171D		1	Yes	Yes	No	[43]
c.613G > C	p.V205L	1		Yes	Yes	**Yes**	
c.637C > T (rs144239448)	p.R213W	3	4	Yes	Yes	No	[36]
c.652G > A	p.A218T		1	Yes	Yes	No	[29]
c.664G > T	p.A222S	1		No	Yes	**Yes**	
c.667C > T	p.R223W	1		Yes	Yes	**Yes**	
c.707G < A	p.G236D	1		Yes	Yes	**Yes**	
c.766C > T	p.P256S	1	1	Yes	Yes	No	[43]
c.801C > A	p.C267X	1		Yes	Yes	**Yes**	
c.820G > A	p.G274S	1		No	Yes	No	[44]
c.832A > G	p.K278E	3	1	Yes	Yes	No	[24]
c.842A > G (rs141177570)	p.N281S		1	Yes	Yes	No	[45]
c.853G > A	p.A285T	1		No	Yes	**Yes**	
c.854C > G	p.A285G	1		Yes	Yes	**Yes**	
c.861C > G	p.I287M	2		Yes	Yes	No	[46]
c.865T > C	p.C289R		1	Yes	Yes	No	[47]
c.880G > C (rs1805009)	p.D294H	85	35	RHC	No	No	[27]
c.892T > C	p.Y298H	1		Yes	Yes	No	[42]
c.895G > A	p.A299T	1		Yes	Yes	No	[25]
c.917G > A	p.R306H	1		Yes	Yes	No	[45]
c.928A > C	p.K310Q	1		No	Yes	**Yes**	
c.951G > T	p.W317C		1	Yes	Yes	**Yes**	
Insertion/deletion							
c.86_87 insA		4		Yes	Yes	No	[46]
c.481_482 insG		1		Yes	Yes	**Yes**	
c.524_525 insT		1		Yes	Yes	**Yes**	
Synonymous							
c.366G > A	p.V122V	1		No	Yes	No	[29]
c.414C > T	p.I138I		1	No	Yes	No	[38]
c.426C > A	p.R142R		2	No	Yes	**Yes**	
c.471C > T	p.T157T		1	No	Yes	**Yes**	
c.477G > C	p.P159P		1	No	Yes	**Yes**	
c.478C > A	p.R160R	1		No	Yes	No	[48]
c.483G > A	p.A161A		1	No	Yes	No	[29]
c.504C > T (rs34612847)	p.I168I		1	No	Yes	No	[38]
c.531G > A (rs145781072)	p.T177T	1	1	No	Yes	No	
c.537C > T	p.F179F		1	No	Yes	**Yes**	
c.621C > T	p.Y207Y		1	No	Yes	No	[31]
c.690G > C	p.P230P		3	No	Yes	No	[24]
c.699G > A (rs146544450)	p.Q233Q	6	10	No	Yes	No	[46]
c.792C > T	p.I264I		4	No	Yes	No	[49]
c.828C > T	p.I276I		1	No	Yes	No	[31]
c.873C > T	p.A291A	1		No	Yes	**Yes**	
c.894C > T (rs143395134)	p.Y298Y		2	No	Yes	No	
c.900C > T (rs3212367)	p.F300F	1	4	No	Yes	No	[38]
c.927C < G	p.L309L	1		No	Yes	**Yes**	
**c.942A > G (rs2228478)**	**p.T314T**	249	170	No	No	No	[25]
c.948C > T (rs151318945)	p.S316S	5	5	No	Yes	No	dbSNP

^a^Damaging variants were those predicted as deleterious or intolerated by SIFT, SNPs3D, and PolyPhen *in silico* prediction tools.

^b^Rare variants were defined as allele frequency less than 1%.

^c^Variants were absent in dbSNP by using NM_002386.3 as contig transcript and works of Gerstenblith et al. [22] and García-Borrón et al. [23].

RHC, red hair colour variant.

Frequent non-RHC variants are shown in bold.

**Table 2:** Association of different variant subgroups with melanoma risk.

	Patients^c^ (chr = 2262)	Controls^c^ (chr = 1738)	OR [95% CI]	*P* value	PAF (%)
Reference sequence^a^	936	976	Ref.	Ref.	
All *R* variants^b^	500/0.22	196/0.11	**2.66 [2.20–3.23]**	2.69**E** − 25	**15.8**
Individual RHC variant					
D84E	23/0.01	13/0.007	1.85 [0.89–3.87]	0.092	0.8
R142H	29/0.01	13/0.007	**2.33 [1.16–4.75]**	**0.012**	**1**
R151C	211/0.09	76/0.04	**2.90 [2.18–3.86]**	7.95**E** − 15	**7.4**
R160W	152/0.07	59/0.03	**2.69 [1.94–3.72]**	1.60**E** − 10	**5.7**
D294H	85/0.04	35/0.02	**2.53 [1.66–3.87]**	3.10**E** − 06	**2.9**
All *r* variants	980/0.43	678/0.39	**1.51 [1.32–1.73]**	1.40**E** − 09	**16.6**
Frequent *r* variants					
V60L	359/0.16	254/0.15	**1.47 [1.22–1.78]**	3.50**E** − 05	**6.5**
V92M	188/0.08	113/0.07	**1.73 [1.34–2.25]**	1.30**E** − 05	**4.5**
I155T	35/0.02	14/0.008	**2.61 [1.35–5.12]**	**0.002**	**1.3**
R163Q	75/0.03	57/0.03	1.37 [0.95–1.98]	0.087	1.2
Rare *r* variants	57/0.025	31/0.017	**1.92 [1.20–3.07]**	**0.004**	**1.6**
*D* variants	48/0.02	21/0.01	**2.38 [1.38–4.15]**	**0.001**	**1.6**
*nD* variants	9/0.004	10/0.006	0.94 [0.35–2.51]	1	

^a^Number of alleles containing wild-type sequence or synonymous variants and used as reference.

^b^All *R* variants including D84E, R142H, R151C, R160W, and D294H.

^c^Number of alleles/MAF (minor allelic frequency).

*R*: red hair colour variant; *r*: nonred hair colour variant; *D*: predicted to be damaging; *nD*: predicted to be nondamaging; OR: odds ratio; CI: confidence interval; PAF: population attributable fraction.

Statistically significant results are shown in bold. Ref, used as reference.

**Table:** (a) *R* variants

	OR [95% CI]	*P* value^a^
Hair colour	0.88 [0.69–1.12]	0.292
Eye colour	**2.30 [1.63–3.26]**	2.64**E** − 06
Skin type	**1.64 [1.17–2.3]**	**0.004**
Nevus count	1.39 [0.97–1.98]	0.07
* R* variants	**2.22 [1.66–2.97]**	6.00**E** − 08

**Table:** (b) *r* variants

	OR [95% CI]	*P* value^a^
Hair colour	0.98 [0.74–1.29]	0.88
Eye colour	**1.93 [1.45–2.57]**	1.20**E** − 06
Skin type	**1.96 [1.49–2.57]**	7.33**E** − 06
Nevus count	**1.6 [1.18–2.15]**	**0.002**
* r* variants	**1.26 [1.04–1.52]**	**0.018**

^a^ *P* value was calculated by logistic regression.

*R*: red hair colour variant;* r*: nonred hair colour variant.

Statistically significant results are shown in bold.

**Table 4:** Localisation of *MC1R* variants according to their classification and to their demonstrated or predicted functional impact.

	Protein region	RHC variant (*R*)	Non-RHC variant (*r*)		
			Frequent non-RHC (*r) *	Rare *r *	
				Predicted to be damaging (*D*)^a^	Predicted to be nondamaging (*nD*)^a^
N-terminus	M1-E37			1	1
Transmembrane 1	V38-I63		x	4	5
Intracellular 1	A64-P72			3	2
**Transmembrane 2**	M73-L100	x	x	16	2
Extracellular 1	L101-Q115			0	1
Transmembrane 3	L116-Y143	x		7	6
**Intracellular 2**	I144-R162	x	x	4	0
Transmembrane 4	R163-Y183		x	1	0
Extracellular 2	D184-L192			0	0
**Transmembrane 5**	V193-A218			9	0
Intracellular 3	Q219-G236			2	1
Transmembrane 6	L237-V265			2	0
Extracellular 3	L266-I276			1	1
**Transmembrane 7**	F277-A299	x		11	2
C-terminus	F300-W317			2	1

^a^Number of alleles found in each protein domain.

The crosses indicate localisation of RHC variants or frequent non-RHC variants.
