# Supplementary material for: A Large French Case-Control Study Emphasizes the Role of Rare Mc1R Variants in Melanoma Risk
Source: Biomed Res Int. 2014 Apr 10;2014:925716. doi: 10.1155/2014/925716 (PMC4003837; doi:10.1155/2014/925716)
Supplement: Supplementary file 1 — Supplementary Table 1: Pigmentation characteristics, which include hair, eye, and skin color, skin type, nevus count, and heavy freckles, were collected from patients and controls studied in this work. Supplementary table 2: Functional impact of all MC1R variants, which have been investigated to date, was collected from many different studies and summarized in this table. [file 925716.f1.pdf]

Supplementary table 1 Pigmentation characteristics of patients and controls

		Patients n=1131 (%)	Controls n=869 (%)	*P*-value[a]
**Hair colour**	Dark	389 (34)	420 (48)	Ref.
	Light	585 (52)	428 (49)	**4.75E-05**
	NA	157 (14)	21 (3)	
**Eye colour**	Dark	319 (28)	372 (43)	Ref.
	Light	579 (51)	294 (34)	**2.54E-14**
	NA	233 (21)	203 (23)	
**Skin colour**	Dark	58 (5)	65 (8)	Ref.
	Light	900 (80)	775 (89)	0.162
	NA	173 (15)	29 (3)	
**Skin type**	III-IV	435 (38)	565 (65)	Ref.
	I-II	586 (52)	286 (33)	**4.11E-14**
	NA	110 (10)	18 (2)	
**Nevus count**	≤50	687 (61)	657 (76)	Ref.
	>50	293 (26)	157 (18)	**2.5E-07**
	NA	151 (13)	55 (6)	
**Heavy freckles**	No	560 (50)	630 (73)	Ref.
	Yes	328 (29)	212 (24)	**1.5E-07**
	NA	243 (21)	27 (3)	

[a] *P* values have been calculated with Fisher exact test.

Abbreviations: NA, not available; Ref, used as reference.

Statistically significant results are shown in bold.

Supplementary table 2 Functional effect of *MC1R* variants

Nucleotide change	Amino acid change	Functional effect	Reference
Non-synonymous			
c.100C>T	p.R34W	ND	
c.104G>A	p.C35Y	Likely total loss-of-function	(Fargnoli et al., 2003; Zanna et al., 2008)
c.112G>A	p.V38M	ND	
c.122C>G	p.S41C	Reduced receptor binding and coupling; moderate intracellular retention	(Fargnoli et al., 2003; Oliva et al., 2009)
c.133T>C (rs61996344)	p.F45L	ND	
c.178G>T (rs1805005)	p.V60L	Reduced receptor binding and coupling; moderate intracellular retention	(Beaumont et al., 2007; Box et al., 1997; Schioth et al., 1999)
c.199C>T	p.R67W	ND	
c.200G>A (rs34090186)	p.R67Q	ND	
c.205C>G	p.L69V	ND	
c.241G>C	p.A81P	ND	
c.247T>C (rs34474212)	p.S83P	ND	
c.252C>A (rs1805006)	p.D84E	Absent receptor binding and coupling; strong intracellular retention	(Beaumont et al., 2007; Valverde et al., 1995)
c.274G>A	p.V92M	Normal	(Beaumont et al., 2005; Koppula et al., 1997; Valverde et al., 1995)

c.284C>T (rs34158934)	p.T95M	ND	
c.296T>C	p.L99P	ND	
c.310G>A (rs2229617)	p.G104S	ND	
c.350A>T	p.D117V	ND	
c.359T>C (rs33932559)	p.I120T	ND	
c.364G>A	p.V122M	Reduced receptor binding and coupling	(Jimenez-Cervantes et al., 2001)
c.373T>C	p.C125R	ND	
c.389C>T	p.S130F	ND	
c.415G>A	p.A139T	ND	
c.417G>A	p.V140M	ND	
c.419T>G	p.V140G	ND	
c.424C>T	p.R142C	ND	
c.425G>A (rs11547464)	p.R142H	Reduced receptor coupling	(Beaumont et al., 2007; Box et al., 1997; Schioth et al., 1999)
c.451C>T (rs1805007)	p.R151C	Reduced receptor binding and coupling; moderate intracellular retention; poor membrane expression	(Beaumont et al., 2007; Box et al., 1997; Schioth et al., 1999; Berta et al ., 2009; Herraiz et al., 2009; Sanchez Mas et al., 2002)
c.456C>A	p.Y152X	Likely total loss-of-function	(John and Ramsay 2002)
c.464T>C (rs1110400)	p.I155T	Absent receptor binding and coupling; strong intracellular retention	(Beaumont et al., 2007; Box et al., 1997)

c.467T>C	p.V156A	ND	
c.478C>T (rs1805008)	p.R160W	Reduced receptor binding and coupling; moderate intracellular retention; poor membrane expression	(Beaumont et al., 2005; Box et al., 1997; Schioth et al., 1999; Berta et al., 2009; Herraiz et al., 2009)
c.479G>A	p.R160Q	ND	
c.488G>A (rs885479)	p.R163Q	Reduced receptor binding and coupling; moderate intracellular retention	(Beaumont et al., 2007; Box et al., 1997)
c.512C>A	p.A171D	ND	
c.613G>C	p.V205L	ND	
c.637C>T (rs144239448)	p.R213W	ND	
c.652G>A	p.A218T	ND	
c.664G>T	p.A222S	ND	
c.667C>T	p.R223W	ND	
c.707G<A	p.G236D	ND	
c.766C>T	p.P256S	ND	
c.801C>A	p.C267X	ND	
c.820G>A	p.G274S	ND	
c.832A>G	p.K278E	ND	
c.842A>G (rs141177570)	p.N281S	Normal	(Liboulet et al., 2006; Oliva et al., 2009)
c.853G>A	p.A285T	ND	

c.854C>G	p.A285G	ND	
c.861C>G	p.I287M	ND	
c.865T>C	p.C289R	Absent receptor binding and coupling	(Oliva et al., 2009)
c.880G>C (rs1805009)	p.D294H	Increased receptor binding; absent receptor coupling; plasma membrane accumulation	(Beaumont et al., 2005; Schioth et al., 1999; Valverde et al., 1995; Berta et al., 2009; Herraiz et al., 2009)
c.892T>C	p.Y298H	ND	
c.895G>A	p.A299T	ND	
c.917G>A	p.R306H	ND	
c.928A>C	p.K310Q	ND	
c.951G>T	p.W317C	ND	

Abbreviation: ND; not determined
